# Admission Outcome and Antimicrobial Resistance Pattern of Bacterial Isolates among Neonates with Suspected Sepsis in Neonatal Intensive Care Unit at Dessie Comprehensive Specialized Hospital, Dessie, Northeastern Ethiopia

**DOI:** 10.1155/2022/1318295

**Published:** 2022-07-08

**Authors:** Genet Molla Fenta, Hiwot Ketema Woldemariam, Yeshi Metaferia, Abdurahaman Seid, Daniel Gebretsadik

**Affiliations:** ^1^Department of Medical Laboratory Science, College of Medicine and Health Sciences, Wollo University, P.O. Box 1145, Dessie, Ethiopia; ^2^Ethiopia Public Health Institute, Addis Ababa, Ethiopia

## Abstract

**Background:**

Neonatal sepsis is a major cause of morbidity and mortality globally. The aim of this study was to assess admission outcome and antimicrobial susceptibility pattern of bacterial isolates among neonates with suspected sepsis at the Dessie Comprehensive specialized Hospital (DCSH), Northeastern Ethiopia.

**Method:**

Cross-sectional study was conducted from August 2017 to March 2018. Two hundred forty-six neonates were recruited, and each patient's blood specimen was collected aseptically using bottle containing Brain Heart Infusion for blood culture. Both clinical and laboratory data such as bacterial culture growth and antimicrobial susceptibility pattern were collected from the neonate; clinical data from the mothers were also included. Antimicrobial susceptibility testing was performed using Kirby-Bauer disk diffusion method. The data were analyzed using SPSS version 20.

**Results:**

Bacteria were identified from 67 (27.2%) blood cultures. The predominant pathogen was *Escherichia coli* (35.8%) followed by *Staphylococcus aureus* (26.8%), and Coagulase Negative *Staphylococcus* (CoNS) (19.4%). The isolated bacteria showed resistance to Ampicillin 55 (82%), third-generation Cephalosporins 21 (58.3%) and other tested antimicrobials. Overall, 68.6% bacterial isolates demonstrated Multidrug resistance (MDR) and total registered mortality rate was 12/246 (4.8%). Both neonatal factors such as neonatal temperature, septic umbilicus and utilization of indwelling medical device during delivery; and maternal factors such as age, antenatal urinary tract infection (UTI), mode of delivery and prolonged rupture of membrane (PROM) had shown statistically significant association with bacterial sepsis.

**Conclusion:**

The rate of bacterial growth was found to be high; *E. coli* and *S. aureus* were the predominant organisms. Both maternal and neonatal related data were strong predictors for bacterial infection of the neonate. Therefore, improving infrastructures for screening of bacteremia as well as active surveillance in clinical setting needed to ensure proper empirical therapy.

## 1. Introduction

Neonatal sepsis is a major cause of morbidity and mortality; the third leading cause of mortality globally [1], and more than half sepsis-related deaths occurred within 28-days of life in developing countries [2]. According to Ethiopian Federal Ministry of Health report (2016), in Ethiopia one neonate died in every 35 children within the first months of life [3]. Moreover, neonatal sepsis is the most common clinical diagnosis and second cause of admission in neonatal intensive-care unit (NICU), in Ethiopia [4].

Neonatal sepsis is characterized by a systemic condition of bacterial, viral, or fungal origin that is related to hemodynamic changes and other clinical manifestations during the primary month of life [5, 6]. Early onset neonatal sepsis (EOS) is a clinical manifestation that occurs within 3 days of life while late-onset neonatal sepsis (LOS) occurs after 3 days of life [4, 6].

Neonates are easily suffering from infectious disease due to their immature components of immune system [5, 7]. Bacterial agents are widely studied in the incidence of neonatal sepsis globally. The leading cause of bacteremia in low and middle income countries were *Staphylococcus aureus*, *Escherichia coli* and *Klebsiella* species [6, 8–10].

Multidrug resistant (MDR) bacterial infections are a major global problem and an estimated, 30% of all global neonatal sepsis mortality is due to MDR pathogens [11]. Various studies have indicated most pathogenic bacteria were resistant to empirical therapy in the management of neonatal sepsis, and the rate of mortality due to MDR bacteria increased in developing countries [9, 12–15]. In particular, neonates in the intensive-care unit were more prone to acquire MDR pathogens due to different risk factors and underlying conditions like pre-maturity, exposure to invasive device, use of antibiotics and colonization of intestinal Gram-negative pathogens [10, 16, 17].

Bacterial infections and their antimicrobial susceptibility patterns were varying from place to place; even within the same place at different duration of time [8, 18, 19]. But still the data reported so far in regards to prevalence, type of bacterial isolate and their antimicrobial resistance pattern were limited and varied in several areas [8, 20]. Neonatal sepsis is also a life threatening emergency, if delaying in diagnosis and treatment with appropriate antibiotics might have unwelcome consequences for the lives of neonates. Therefore, this study aims to assess admission outcome and antimicrobial susceptibility pattern of bacterial isolates among neonates with suspected sepsis who were admitted in NICU at Dessie Comprehensive Specialized Hospital (DCSH), Northeastern Ethiopia.

## 2. Materials and Methods

### 2.1. Study Setting and Study Design

An institution based cross-sectional study was carried out from August 2017 to March 2018 at DCSH, Dessie, South Wollo Zone, Amhara region. Dessie is located at the latitude of 11^o^8′N and longitude of 39^o^38′E with an elevation between 2,400 and 3,200 meters above sea level. The town is found 401 km distant from the capital city of Ethiopia (Addis Ababa). The hospital catchment population is about 7 million, and in 2016 the entire outpatient attendants were 205,454. The NICU is part of the pediatric department wards and the average total number of neonates' admissions per year is almost 1600. In addition to the populations in Desssie town, the hospital also serves as a referral site for neighboring North Wollo, Oromiya zone of Amhara regional state, and Afar regional state.

### 2.2. Study Population

**Figure 1:**
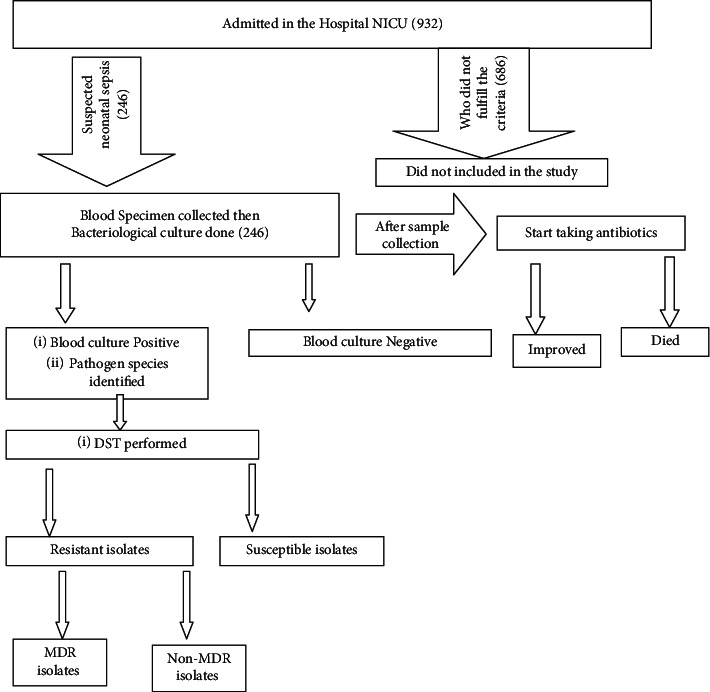
Flow chart illustrating study population, bacteriologic activities and treatment outcome in NICU at Dessie comprehensive specialized hospital during the study period.

In this study, the clinical diagnosis of sepsis was made by considering the Integrated Management of Neonatal and Childhood Illness (IMNCI) guideline [21]. All neonates presented with one or more clinical features found in IMNCI guideline and who were admitted to DCSH-NICU during the study period were the study populations. Neonates admitted to NICU and whose mothers and/or caregivers provided written consent were included in this study. Neonates who showed gross congenital anomalies, severe anemia and those who received antibiotics prior to blood specimen collection were not included. During the study period, a total of 932 neonates were admitted with various types of clinical diagnosis. Of these, a total of 246 neonates who fulfilled the above inclusion criteria were enrolled consecutively (Figure 1).

### 2.3. Study Variables

In this study, the dependent variable was the presence of bacterial growth (infection), whereas independent variables were maternal and neonatal characteristics.

### 2.4. Data Collection

This study uses data that were collected from both structured questionnaire and from bacteriological laboratory work results of blood culture. Socio-demographic characteristics and other associated variables of each delivered mother and clinical data of their neonate were collected and extracted from their medical history.

### 2.5. Specimen Collection and Processing

From each study subject, 2.5 ml of venous blood was collected aseptically and transferred into double 20 ml of Brain heart infusion blood culture bottle. After collection, all blood specimens were incubated at 35–37°C in the DCSH laboratory until transported to the bacteriology laboratory of the Amhara Public Health Institute (APHI), Dessie branch.

### 2.6. Bacteriological Culture and Identification

All blood culture bottles were incubated at 35–37°C under aerobic condition for 7 days. According to the standard operating procedures (SOPs) the incubated blood culture bottles were sub-cultured on Blood agar, Chocolate agar and MacConkey agar. The inoculated Blood agar (Oxoid Ltd.) and MacConkey agar (Oxoid Ltd. Basingstoke, Hampshire, UK) plates were incubated aerobically at 35–37°C for 24 hours. Whereas, blood specimen sub-cultured on Chocolate agar (Oxoid Ltd.) plates were incubated at 35–37°C in microaerophilic condition (5–10% CO_2_) for 48–72 hours. If CoNS was isolated from a single blood culture bottle, then it was considered as contaminant growth, whereas if it was isolated from two blood culture bottles it was considered as a pathogen [22].

Each bacterial isolate was identified through colony morphology, Gram stain, biochemical test and rapid bench test like catalase and oxidase test. Biochemical tests (Indole production, motility, citrate utilization, urea, triple sugar iron, and decarboxylase/lysine iron agar/test) using conventional methods were performed for Gram-negative isolates. In addition to rapid bench tests, other tests (PYR-test, Optochin, coagulase, Bile solubility, Bile-Esculin, DNAase) were also used to differentiate Gram-positive isolates [22].

### 2.7. Antimicrobial Susceptibility Testing

Antimicrobial susceptibility testing was performed for each bacterial isolate using a modified Kirby-Bauer disk diffusion method as per Clinical and Laboratory Standards Institute (CLSI) guideline [23]. Using a sterile wire loop, 3–5 well isolated colonies were picked from the primary inoculated plates. The suspended colony was adjusted for the inoculums' turbidity equivalent to 0.5 McFarland standard and swabbed on Mueller Hinton Agar (MHA) (Conda Ltd, USA) plate. Standard antibiotic disks were placed aseptically and then the inoculated MHA plate was incubated at 35–37°C for 16–18 hours. In this study the utilized antibiotic concentration per disk (Oxoid UK and BBL-BD USA) were PenicillinG-10 unit, Ampicillin-10 *μ*g, Gentamicin-10 *μ*g, Clindamycin-2 *μ*g, Erythromycin-15 *μ*g, Trimethoprim-sulfamethoxazole-1.25/23.75 *μ*g, Cefoxitin-30 *μ*g, Amoxicillin-Clavulanic acid-20/10 *μ*g, Piperacillin-Tazobactam 100 *μ*g/10 *μ*g, Ceftazidime-30 *μ*g, Ceftriaxone-30 *μ*g, Cefotaxime-30 *μ*g, Cefepime-30 *μ*g, and Meropenem-10 *μ*g. The zone of inhibition for the above-mentioned antibiotics, with the exception of Cefoxitin, was measured after 16–18 hours of incubation. Based on the CLSI guideline [23], the results obtained from the antimicrobial susceptibility testing were interpreted as susceptible, intermediate and resistance. Multidrug resistance was interpreted as resistance to a minimum of three or more antibiotic classes [24]; intermediate results in this study were categorized as resistance.

### 2.8. Quality Control

By considering both CLSI guideline and the SOPs of the medical microbiology laboratory unit at Amhara public health institute, all standard procedures in the three important phases were strictly followed. The sterility of culture media was ensured by incubating 5% of each batch of the prepared media at 37°c for 24 hours. In order to assure the performance of all prepared media, we have used standard-strains like *Escherichia coli* (ATCC)-25922, *Pseudomonas aeruginosa* (ATCC-27853), *Staphylococcus aureus* (ATCC-25923), *Haemophilus influenzae* (ATCC-49247), *Streptococcus pneumoniae* (ATCC-49029) and *Enterococcus fecalis* (ATCC-29212).

### 2.9. Data Processing and Analysis

The data were checked for their completeness and analyzed using SPSS version 20. Frequencies and cross tabulations accustomed to summarize descriptive statistics. Bi-variable and multi-variable logistic regressions were used to assess the association between dependent and independent variables. The odds ratio and 95% confidence interval were computed to assess the presence and degree of association between dependent and independent variables. The *P* -value < 0.05 was considered statistically significant.

### 2.10. Ethical Considerations

Ethical clearance was obtained from an institutional ethical review committee of the College of Medicine and Health Science, Wollo University. Additionally, the official permission letter was secured from DCSH using the cooperation letter that was written by Wollo University. After briefly explaining the importance, purpose and procedure of the study, a written assent form was obtained from each study participant's family.

## 3. Results

### 3.1. Characteristics of Study Participants

A total of two hundred forty-six neonates with suspected sepsis participated. Out of the total number of participating neonates, 82.5% (203/246) were suspected of early onset sepsis; 66% (162/246) of them were male; 48.4% (119/246) had low birth weight (<2500 gm) and 45% (111/246) of neonates were preterm. Regarding the clinical characteristics of the neonate; 75 of them showed lethargy, 201 neonates refused to feed, 10 study participants had septic umbilicus and 37 of them had experienced vomiting (Table 1).

### 3.2. Maternal Related Information

About 197 (80%) mothers were below 35 years old, 219 (89%) were married and 134 (54.47%) were urban area inhabitants. Most of the mothers delivered in the health facilities, the majority of them gave birth through spontaneous vortex delivery and 8 (3.25%) mothers experienced urinary tract infection (UTI) during their pregnancy period (Table 2).

### 3.3. Blood Culture Findings

Bacterial growth obtained from 27.2% (67/246) of the total participated neonates and among the overall identified bacterial pathogens near to two-thirds was obtained from neonates with EOS. Bacterial growth was detected among 17% (42/246) of male and 10.2% (25/246) of female study participants. Furthermore, bacterial isolates were identified from 15.8% (39/246) of neonates born preterm and 16.7% (41/246) of neonates with low birth weight (Table 1). The majority (89.55%) of bacterial growth obtained among neonates who were delivered from currently married mothers and about 74.6% of neonates with bacterial growth result were born from mothers whose age was below 35 years (Table 2).

### 3.4. Associated Factors for Bacterial Growth

To verify the presence or absence of an association between dependent and independent variables, the logistic regression analysis of data from both neonates and mothers were analyzed. Study participated neonates who had septic umbilicus showed 10 times higher odds of developing bacterial infection in comparison with those neonates who had clear umbilicus [AOR = 10.06, 95%CI = 1.75–57.8, *P*=0.01]. Utilization of an indwelling medical devices has shown 2 times higher likelihood of bacterial infection among the sepsis suspected neonates [AOR = 2.2, 95%CI = 1.12–4.32, *P*=0.022] (Table 3). Neonates who were born from mothers whose age was less than 35 years had a 57% reduced likelihood of acquiring bacterial infection (AOR = 0.43, 95%CI = 0.2–0.92, *P*=0.029). Neonate delivered from pregnant mother with UTI had 19 times higher odds (AOR = 19, 95%CI = 2.02–178.7, *P*=0.01) of bacterial infection to their counterparts (Table 4).

### 3.5. Isolated Bacteria

**Figure 2:**
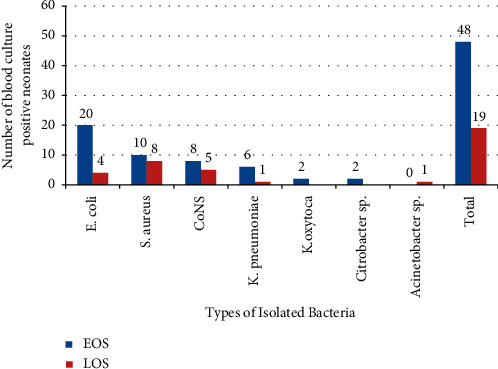
Frequency and types of organisms from neonates admitted in NICU at Dessie comprehensive specialized hospital during the study period.

From the total positive blood culture, 53.7% (36/67) were found to be Gram-negative and seven different types of bacterial pathogens were identified. The most frequently isolated bacterial species was *E. coli,* 35.8% (24/67), followed by *S. aureus,* 26.8% (18/67) and CoNS, 19.4% (13/67). Whereas *K. pneumoniae,* 10.4% (7/67), *K. oxytoca,* 2.9% (2/67), Citrobacter sp, 2.9% (2/67) and Acinetobacter sp, 1.4% (1/67) were the least frequent isolates. Most, 83.3% (30/36), Gram-negative bacteria were identified from study participants who had EOS (Figure 2).

### 3.6. Antimicrobial Susceptibility Pattern of Isolated Bacteria

From the total bacterial isolates, 56 (83.5%) were resistance to one or more antibiotics. Out of these resistant isolates, 26 (46.4%) were observed among Gram-positive bacteria while 30 (53.5%) were among Gram-negative bacteria. Higher levels of resistance to Penicillin/Ampicillin, 26/31 (83.87%); Cefoxitin, 22/31(71%) and Trimethoprim-Sulfamethoxazole, 21/31 (67.7%) while lower level of resistance to Clindamycin, 2 (6.45%) and Gentamicin, 8 (25.8%) was observed among Gram-positive isolates. Moreover, most of the Gram-negative bacteria were resistance to Ampicillin, 29 (82.9%); Amoxicillin-Clavulanate, 27 (77%); Trimethoprim-Sulfamethoxazole, 26 (72.2%); Piperacillin-Tazobactam, 23 (63.8%); third-generation Cephalosporins, 21 (58.3%) and Gentamicin, 16 (44.4%). The majority (86%) of Gram-negative isolates were susceptible to Meropenem (Table 5).

Overall 55 (82%) and 24 (35.8%) isolates were resistance to Ampicillin and Gentamicin, respectively. More than half *S. aureus* isolates, 12 (66.7%) were found to be Methicillin resistant *Staphylococcus aureus* (MRSA). In addition, 52.4% *E. coli* isolates and 28.6% *Klebsiella* species were found to develop resistance to third-generation Cephalosporins. Gram-negative isolates demonstrated antibiotic resistances ranging from 13.9% to 82.8% for certain drugs like Carbapenems, Aminoglycosides, Cephalosporins, Trimethoprim-Sulfamethoxazole, Piperacillin-Tazobactam, Amoxacillin-Clavulanate and Ampicillin. Gram-positive isolates had exhibited antibiotic resistance rate between 6% and 83.8% against Lincosamides, Aminoglycosides, Macrolides, Trimethoprim-Sulfamethoxazole, Cefoxitin and Penicillins (Table 5).

### 3.7. Multidrug Resistant Bacterial Isolates

In the current study, 68.6% (46/67) of bacterial isolates exhibited MDR and out of the total MDR isolates Gram-negative bacteria accounted for 52.2% (24/46) while 47.8% (22/46) were Gram-positive bacteria. Moreover, 85.7% (6/7) of *K. pneumonia*e, 62.5% (15/24) of *E. coli* and 66.7% (12/18) of *S. aureus* were found to be MDR (Table 6).

### 3.8. Admission Outcome in Neonates with Suspected Sepsis

From the total number of participating neonates, 234 (95.2%) were improved to discharge, whereas 12 (4.8%) neonates were died before receiving their complete dosage of antibacterial therapy. Among the total neonates with death outcome 7 were developing EOS and the rest 5 were having LOS. Out of died neonates with EOS condition, 4 were having negative blood culture result and from neonates having death outcome with LOS, 3 were found to be infected by bacterial pathogen. Six (50%) died neonates were having positive blood culture; the bacterial isolated from these study participants were *E. coli* [3], *K. pneumoniae* [2] and *S. aureus* [1]. Most bacteria that were isolated from neonates with death outcome showed higher resistance pattern to virtually all tested antibiotics; only 2 isolates were susceptible to Gentamicin, Meropenem and Piperacillin-Tazobactam (Table 7).

The majority of neonates (231) received Ampicillin plus Gentamicin, thirteen neonates received Ampicillin plus Cefotaxime and the rest 2 received Ceftazidime plus Vancomycin. Among six neonates who had death outcome and had negative blood culture result, four were receiving Ampicillin plus Gentamicin and the remaining two were receiving Ampicillin plus Cefotaxime. Among 6 neonates who had positive blood culture result with death outcome, two had received Ampicillin plus Gentamicin; one had received Ceftazidime plus Vancomycin and three had received Ampicillin plus Cefotaxime.

## 4. Discussion

In the present study, the overall blood culture positive rate (27.2%) was in agreement with similar studies conducted in Bangladesh (28.4%) [25], Indonesia (24.6%) [26] and Assela, Ethiopia (29.4%) [20]. In contrast, it was lower than studies done in Gondar, Ethiopia (46.6%) [8], Egypt (40.7%) [27] and India (41.7%) [28]. It was also higher than studies done in Botswana (9.8%) [29] and Ghana (17.3%) [30]. Furthermore, a higher proportion of bacterial isolates among EOS were observed in the present study, which was in agreement with studies conducted in Ghana [30] and Gondar, Ethiopia [8]. But a paradoxical result was reported from Tanzania [9]. The difference in proportion of isolated bacterial pathogen across studies might be due to methodological variation and variation in study settings.

According to the results of the present study the proportions of bacterial isolates were higher among low birth weight and preterm neonates; which was similar to other previously conducted studies [8, 19, 27]. Preterm and low birth weight is well-known associated risk factors in the occurrence of bacterial sepsis in the newborn. This is due to limited capacity in cellular innate immune system with inflammatory response factors following infections [5, 7]. However, in the current study there was no statistical significant association between blood culture positive results with the birth weight and gestational age.

In this research work, the proportion of Gram-positive and Gram-negative bacterial isolates were comparable with other findings reported from African and Asian countries [18, 20, 25–27, 31–34]. The current study revealed that *E. coli* and *S. aureus* were the predominant isolates; this was in line with studies done in different parts of Ethiopia like Gondar, [8], Addis Ababa [19], Assella [20] and other developing countries [14, 25, 31]. *E. coli* is a member of normal microbiota found in the gastrointestinal tract, and associated with severe infections and meningitis in neonates. Following a normal vaginal birth, newborns can be frequently colonized with maternal vaginal and fecal flora, hence, newborns can acquire during or before delivery [5, 35]. Moreover, *S. aureus* is found as communal bacterium (skin and nose); newborns can easily be contaminated from their mothers during or after delivery and acquire staphylococcal infections [5, 35].

A lower proportion of *K. pneumoniae* (10.4%) was observed in this study, which was comparable with studies done in different localities of Ethiopia [8, 19, 20] and in some developing countries [26, 28, 30, 31, 36, 37]. This bacterium is an important pathogen causing hospital-acquired bloodstream infections, particularly the ESBL-producing *K. pneumoniae* species was found to be the most important contributor for the emergency of MDR threat [10, 38]. On the contrary to the present study, Kabwe et al. reported *Klebsiella* species as the predominant isolates identified among neonatal sepsis suspected populations in Zambia.

In the present study, most of the isolated bacteria showed resistance to commonly used antibiotics; which was in agreement with studies reported from other developing countries [8, 9, 18–20, 26, 27, 37, 39]. Low level resistance to Meropenem and Clindamycin were observed among Gram-negative and Gram-positive isolates, respectively. This result was found to be consistent with studies conducted in Gondar, Ethiopia [8], Assella, Ethiopia [20], Asian countries [25, 26, 28] and elsewhere [9, 30, 31, 37]. Meropenem and Clindamycin are mostly prescribed for culture proven ESBL-Gram-negative bacteria and necrotizing enterocolitis with possible perforation, deep tissue infection caused by anaerobic bacteria, respectively. This low rate of resistance might be due to these antibiotics are not routinely prescribed at the study site and/or are currently used as WHO-recommended empirical therapy in the neonatal age group [40].

Ampicillin and Gentamicin are currently used as WHO-recommended first-line empiric therapy [40]. The present study showed there was a higher level of Ampicillin (82%) and moderate level of Gentamicin (35.8%) resistant isolates. It was in accordance with previous findings reported from Gondar, Ethiopia [8, 18], Addis Ababa, Ethiopia [19], Assella, Ethiopia [20] and elsewhere [26, 28, 30, 36, 37, 39]. Ampicillin has broad-spectrum activity and widely used antibiotic and most bacteria developed resistance to this agent. Mechanisms of resistance could be due to overuse of Ampicillin exerted strong selective pressure on bacterial pathogens by increasing the evolution and transfer of genes; favoring the emergence of resistant strains. Moreover, alteration of Penicillin binding proteins and production of beta-lactamases is a mechanism of development of resistance to Gram-positive and Gram-negative bacteria, respectively [38].

This finding indicated the overall MDR level was 68.6%, which was comparable with studies done in Gondar, Ethiopia [8] and Assella, Ethiopia [20]. However, it was lower than other studies reported from Egypt [14] and Equatorial Guinea [36]. Likewise, this research work showed that MDR level in *K. pneumoniae,* 6 (85.7%), and *E. coli,* 15 (62.5%), was comparable with studies done in Gondar, Ethiopia (*K. pneumoniae,* 74%) [8], and Nigeria (62.5% of *K. pneumoniae* and *E. coli)* [39]. However, which was lower than studies conducted in Assella, Ethiopia (*E. coli,* 91%) [20], Equatorial Guinea (*K. pneumoniae,* 100%) [36] and Egypt (*E. coli,* 90%) [14]. Furthermore, 66.7% of *S. aureus* isolates were found to be MRSA, which was correlated with other studies reported from Ethiopia and abroad [8, 14, 18, 20, 39].

In the current finding, we observed a significant number of MDR and MRSA bacterial isolates. Bacteria can develop resistance due to the acquisition of plasmids and due to an innate mechanism of bacterial agents to inactivate antibiotics. Globally, the emergence of strains resistant to the common use of antibiotics increased due to several reasons, such as self-prescription and the overuse of broad-spectrum antibacterial agents. Resistance in *K. pneumoniae* and *E. coli* is usually acquired through plasmid mediated ESBL; the enzymes can destroy beta-lactam antibiotics. These ESBL-associated plasmids can carry genes encoding co-resistance to other antibiotics, resulting MDR phenotype [38]. Methicillin resistant *S. aureus* also carries mecA gene that encode a beta-lactam insensitive protein. Thus, the resistance phenotypes can be disseminated between different bacteria within the same host or another host [38, 41]. Therefore, variation of findings in MDR level might be due to difference in patient population, exposure of invasive procedures; selection of antibiotics from different classes/generations, study setting and studying period.

The current study indicated that the overall proportion of neonatal death was similar with study done in Bahir Dar, Ethiopia (4%) [42], and comparably higher death outcome was reported by Gebrehiwot et al. (8%) [43]. On the contrary to the present study, a much higher mortality rate was reported from Asian and African countries which ranges from 14% to 51% [9, 27, 44, 45]. In the present study, there was no statistical significant difference between death outcome and blood culture result. However, a given study in Ethiopia indicated an association between death outcome and significant bacterial growth [43]. In other way, Gram-negative MDR isolates were found to be higher among neonate with death outcome, which was similar with studies reported from other countries [9, 14, 42–45]. Various studies also indicated that the fatality rate is higher in neonates exposed to bloodstream infections with MDR pathogens compared to non-MDR pathogens [1, 2, 9, 11].

Similar to published article in Ghana [46], the result in the current study had shown both maternal and neonatal factors are responsible for the development of neonatal sepsis. In the present study neonatal data such as presence of septic umbilicus and utilization of indwelling medical devices during delivery had strong statistical association with blood culture positive results. Similar to the current study neonatal sepsis had shown strong association with umbilical pus discharge in a study conducted in Tanzania [47].

In addition to the above-mentioned variables, maternal age below 35 years old, having history of UTI, assisted vaginal delivery and prolonged rupture of membrane greater than or equals to 18 hours are determinants for the bacterial infection among the neonates. Comparably, another study which was conducted in public hospitals in central Ethiopia showed younger maternal age had reduced likelihood of neonatal sepsis [48]. In the current study neonates born to mothers who had antenatal UTI showed 19 times higher odds of acquiring bacterial sepsis in comparison with their counterparts. In agreement with this result, systematic review and meta-analysis in Ethiopia reported that maternal UTI during antenatal care demonstrated remarkable association with the development of neonatal sepsis [49]. Other similar studies in Ethiopia and Bangladesh also exhibited the UTI during pregnancy, prolonged rupture of membrane, place where delivery has done as potential predictor for the occurrence of neonatal septicemia [48, 50, 51].

Similar studies in Ethiopia and Bangladesh indicated that 5^th^ minute APGAR score had shown association with culture confirmed bacteria [20, 50], whereas in the present study both 1^st^ and 5^th^ minute APGAR score did not indicate significant association with bacterial infection.

### 4.1. Limitation of Study

This study was done on hospitalized neonates (NICU) from a single institution, but the results may not be applied to the catchment area or the entire country. Due to unavailability of facilities, Genotypic and specific phenotypic characterization of MDR (ESBL and MRSA) for the isolated bacteria were not performed.

## 5. Conclusion

The prevalence of bacterial growth among neonatal sepsis was found to be high; almost two-third of bacterial growth was detected from neonates with EOS. *E. coli* and *S. aureus* were the predominant organisms. Moreover, almost half of the bacterial isolates showed resistance to multiple antibiotics. Both maternal and neonatal variables were showing statistically significant association with bacterial culture positive result. Therefore, it might be necessary to improve the infrastructures for screening of bacteremia, as well as active surveillance during a clinical setting, to ensure adequate empirical therapy. Further studies should be conducted by using highly sensitive and specific techniques like PCR; phenotypic and genotypic characterization of ESBL, MRSA; active surveillance with large cohort study on neonatal sepsis should be addressed.

## Figures and Tables

**Table 1:** Neonatal data in relation to neonates with suspected sepsis admitted in NICU at Dessie comprehensive specialized hospital, Dessie, Northeastern Ethiopia, August 2017 to March 2018.

S.no.	Characteristics	Number (%) ^*∗*^	
		NG	BG
1	Age in days		
	≤ 3	155 (63%)	48 (19.5)
	>3–28	24 (9.8)	19 (7.7)
2	Gender		
	Female	59 (24)	25 (10.2)
	Male	120 (48.8)	42 (17.1)
3	Birth weight in Gram		
	<2500	89 (35)	33 (13.4)
	≥2500	93 (37.8)	34 (13.8)
4	Neonate gestational age (week)		
	<37	87 (35.4)	33 (13.4)
	≥37	92 (37.4)	34 (13.8)
5.	Birth aspexia		
	No	176 (71.5)	64 (26)
	Yes	3 (1.2)	3 (1.2)
6	Neonatal temperature, (c)		
	35.5–37.5	32 (13)	19 (7.7)
	<35.5	99 (40.2)	17 (6.9)
	>37.5	48 (19.5)	31 (12.6)
7	Respiratory rate		
	≥60	116 (47.2)	35 (14.2)
	40–60	63 (25.6)	32 (13)
8	Pulse rate		
	>160	44 (17.9)	14 (5.7)
	120–160	135 (54.9)	53 (21.5)
9	Lethargy		
	No	127 (51.6)	44 (17.9)
	Yes	52 (21.1)	23 (9.3)
10	Cyanosis		
	No	175 (71.1)	64 (26)
	Yes	4 (1.6)	3 (1.2)
11	Grunting		
	No	134 (54.5)	60 (24.4)
	Yes	45 (18.3)	7 (2.8)
12	Chest in drawing		
	No	171 (69.5)	64 (26)
	Yes	8 (3.3)	3 (1.2)
13	Respiratory distress		
	No	172 (69.9)	64 (26)
	Yes	7 (2.8)	3 (1.2)
14	Feeding		
	Normal	29 (11.8)	16 (6.5)
	Refusal	150 (61)	51 (20.7)
15	Umblicus		
	Clean	177 (72)	59 (24)
	Septic	2 (0.8)	8 (3.3)
16	Clinical jaundice		
	No	169 (68.7)	61 (24.8)
	Yes	10 (4.1)	6 (2.4)
17	Indwelling medical devices		
	No	117 (47.6)	34 (13.8)
	Yes	62 (25.2)	33 (13.4)
18	Vomiting		
	No	156 (63.4)	53 (21.5)
	Yes	23 (9.3)	14 (5.7)
19	Diarrhea		
	No	176 (71.5)	63 (25.6)
	Yes	3 (1.2)	4 (1.6)
20	APGAR score first		
	≥7	163 (66.3)	55 (22.4)
	<7	16 (6.5)	12 (4.9)
21	APGAR score fifth		
	≥7	164 (66.7)	56 (22.8)
	<7	15 (6.1)	11 (4.5)

(%) ^*∗*^: the percentage in each cell is computed by dividing the cell's value (numerator) with the total study subjects (246) (denominator); NG: No growth; BG: Bacterial growth; °c: Degree Celsius.

**Table 2:** Maternal data in relation to neonates with suspected sepsis admitted in NICU at Dessie Comprehensive Specialized Hospital, Dessie, Northeastern Ethiopia, August 2017 to March 2018.

S.no.	Characteristics	Number (%) ^*∗*^	
		NG	BG
1	Age in years		
	<35	147 (59.8)	50 (20.3)
	≥35	32 (13)	17 (6.9)
2	Marital status		
	Single	18 (7.3)	7 (2.8)
	Separated	2 (0.8)	0 (0)
	Married	159 (64.6)	60 (24.4)
3	Residence		
	Rural	80 (32.5)	32 (13)
	Urban	99 (40.2)	35 (14.2)
4	Occupation status of mothers		
	Unemployed	88 (35.8)	29 (11.8)
	Employed	91 (37)	38 (15.4)
5	Educational status		
	Illiterate	21 (8.5)	11 (4.5)
	Primary school level	80 (32.5)	27 (11)
	Secondary and above	78 (31.7)	29 (11.8)
6	No. of parity		
	Primparity	93 (37.8)	36 (14.6)
	2–4	72 (29.3)	26 (10.6)
	≥5	14 (5.7)	5 (2)
7	Number of ANC visit		
	No	1 (0.4)	0 (0)
	<4	87 (35.4)	25 (10.2)
	≥4	91 (37)	42 (17.1)
8	Twin pregnancy		
	No	166 (67.5)	63 (25.6)
	Yes	13 (5.3)	4 (1.6)
9	Urinary tract infection		
	No	178 (72.4)	60 (24.4)
	Yes	1 (0.4)	7 (2.8)
10	Vaginal discharge foul smelling		
	No	174 (70.7)	64 (26)
	Yes	5 (2)	3 (1.2)
11	Place of delivery		
	Hospital	126 (51.2)	43 (17.5)
	Health center	51 (20.7)	24 (9.8)
	Home	2 (0.8)	0 (0)
12	Mode of delivery		
	Spontaneous vortex delivery	142 (57.7)	47 (19.1)
	Assisted vaginal delivery	7 (2.8)	9 (3.7)
	Caesarian section	30 (12.2)	11 (4.5)
13	Duration of labor		
	<18 hrs.	170 (69.1)	62 (25.2)
	≥18 hrs.	9 (3.7)	5 (2)
14	Prolonged rupture of membrane		
	<18 hrs.	160 (65)	39 (15.9)
	≥18 hrs	19 (7.7)	28 (11.4)

(%) ^*∗*^: the percentage in each cell is computed by dividing the cell's value (numerator) with the total study subjects (246) (denominator); NG: No growth; BG: Bacterial growth.

**Table 3:** Bivariable and multi-variable analysis of neonatal data in relation to neonates with suspected sepsis admitted in NICU at Dessie Comprehensive Specialized Hospital, Dessie, Northeastern Ethiopia, August 2017 to March 2018.

S.no.	Characteristics	Bi-variable analysis		Multivariable analysis	
		Corollary (95% CI)	*P*-value	AOR (95% CI)	*P*-value
1	Age in days				
	>3–28	2.56 (1.29–5.06)	0.007	2.15 (0.96–4.8)	0.062
	≤3	Ref		Ref	
2	Gender				
	Female	1.21 (0.67–2.17)	0.522		
	Male	Ref			
3	Birth weight in Gram				
	<2500	1.05 (0.6–1.84)	0.866		
	≥2500	Ref			
4	Neonate gestational age (week)				
	<37	1.03 (0.59–1.8)	0.928		
	≥37	Ref			
5	Birth aspexia				
	No	0.36 (0.07–1.85)	0.223	0.28 (0.04–2.05)	0.211
	Yes	Ref		Ref	
6	Neonatal temperature, °c				
	35.5–37.5	Ref		Ref	
	<35.5	0.92 (0.44–1.9)	0.820	0.26 (0.11–0.65)	0.004^*∗*^
	>37.5	0.27 (0.13–0.53)	0.000	0.82 (0.36–1.88)	0.637
7	Respiratory rate				
	≥60	0.59 (0.34–1.05)	0.073	0.58 (0.29–1.16)	0.123
	40–60	Ref		Ref	
8	Pulse rate				
	>160	0.81(0.41–1.6)	0.545		
	120–160	Ref			
9	Lethargy				
	No	0.78 (0.43–1.42)	0.424		
	Yes	Ref			
10	Cyanosis				
	No	0.49 (0.11–2.24)	0.356		
	Yes	Ref			
11	Grunting				
	No	2.88 (1.23–6.75)	0.015	2.14 (0.82–5.56)	0.119
	Yes	Ref		Ref	
12	Chest in drawing				
	No	0.998 (0.26–3.88)	0.998		
	Yes	Ref			
13	Respiratory distress				
	No	0.87 (0.22–3.46)	0.841		
	Yes	Ref			
14	Feeding				
	Normal	Ref	0.168	Ref	0.794
	Refusal	0.62 (0.31–1.23)		0.9 (0.39–2.04)	
15	Umblicus				
	Clean	Ref	0.002	Ref	0.01^*∗*^
	Septic	12.0 (2.48–58.1)		10.06 (1.75–57.8)	
16	Clinical jaundice				
	No	Ref	0.344		
	Yes	1.66 (0.58–4.77)			
17	Indwelling medical devices				
	No	Ref	0.037	Ref	0.022^*∗*^
	Yes	1.83 (1.04–3.24)		2.2 (1.12–4.32)	
18	Vomiting				
	No	Ref	0.119	Ref	0.350
	Yes	1.79 (0.86–3.73)		1.51 (0.64–3.59)	
19	Diarrhea				
	No	Ref	0.091	Ref	0.574
	Yes	3.72 (0.81–17.11)		1.66 (0.29–9.58)	
20	APGAR score fifth				
	≥7	Ref	0.053	Ref	0.703
	<7	2.22 (0.99–4.99)		1.99 (0.56–68.04)	
21	APGAR score first				
	≥7	Ref	0.073	Ref	0.686
	<7	2.15 (0.93–4.95)		2.04 (0.06–64.01)	

Ref: reference category, ^*∗*^ indicates statistically significantly associated factors; °c: Degree Celsius.

**Table 4:** Logistic regression analysis of maternal data in relation to neonates with suspected sepsis admitted in NICU at Dessie Comprehensive Specialized Hospital, Dessie, Northeastern Ethiopia, August 2017 to March 2018.

S.no.	Characteristics	Bi-variable analysis		Multivariable analysis	
		Corollary (95% CI)	*P*-value	AOR (95% CI)	*P*-value
1	Age in years				
	18–34	0.64 (0.33–1.25)	0.192	0.43 (0.20–0.92)	0.029^*∗*^
	≥35	Ref		Ref	
2	Marital status				
	Currently unmarried	Ref	0.871		
	Currently married	1.08 (0.43–2.68)			
3	Residence				
	Rural	1.13 (0.64–1.99)	0.667		
	Urban	Ref			
4	Occupation status of mothers				
	Unemployed	Ref	0.412		
	Employed	1.27 (0.72–2.23)			
5	Educational status				
	Illiterate	1.41 (0.61–3.28)	0.597		
	Primary school level	0.91 (0.49–1.67)	0.427		
	Secondary and above	Ref	0.756		
6	No. Of parity				
	Primparity	1.08 (0.36–3.23)	0.969		
	2–4	1.01 (0.33–3.08)	0.885		
	≥5	Ref	0.985		
7	Number of ANC visit				
	<4	Ref	0.098	Ref	0.089
	≥4	1.62 (0.91–2.89)		1.79 (0.92–3.5)	
8	Twin pregnancy				
	No	Ref	0.722		
	Yes	0.81 (0.26–2.58)			
9	Urinary tract infection				
	No	Ref	0.005	19 (2.02–178.7)	0.01^*∗*^
	Yes	20.77 (2.5–172)			
10	Vaginal discharge foul smelling				
	No	Ref	0.511		
	Yes	1.63 (0.38–7.02)			
11	Place of delivery				
	Hospital	0.75 (0.42–1.36)	0.350		
	Non-hospital	Ref			
12	Mode of delivery				
	Spontaneous vortex	Ref	0.038	Ref	
	Assisted vaginal delivery	3.88 (1.37–11)	0.011	3.61 (1.05–12.41)	0.042^*∗*^
	Caesarian section	1.11 (0.52–2.38)	0.793	1.45 (0.61–3.42)	0.402
13	Duration of labour				
	<18 hrs	Ref	0.466		
	≥18 hrs.	1.52 (0.49–4.72)			
14	Prolonged rupture of membrane				
	<18 hrs	Ref	0.000	Ref	0.000^*∗*^
	≥18 hrs	6.05 (3.06–11.93)		7.34 (3.44–15.63)	

Ref: reference category, ^*∗*^ indicates statistically significantly associated factors.

**Table 5:** Distribution of antibiotic resistance from blood cultures of isolated bacteria among neonates with suspected sepsis admitted in NICU at Dessie Comprehensive Specialized Hospital, Dessie, Northeastern Ethiopia, August, 2017 to March, 2018.

Gram-negative bacteria	Antibiotic resistance pattern, *n* (%)									
	AMP	AMC	PTZ	CPM	CAZ	Cxt	CRO	CN	SXT	MEM
*E. coli* (24)	19 (79.2)	18 (75)	15 (65.6)	11 (45.8)	13 (54.2)	14 (58.3)	14 (58.3)	10 (41.7)	16 (66.7)	2 (8.3)
*K. pneumoniae* (7)	7 (100)	6 (86)	5 (71.4)	5 (71.4)	5 (71.4)	5 (71.4)	5 (71.4)	4 (57)	7 (100)	1 (14.3)
*K. oxytoca* (2)	2 (100)	2 (100)	1 (50)	1 (50)	1 (50)	1 (50)	1 (50)	1 (50)	1 (50)	1(50)
Citrobacter sp (2)	1 (50)	1 (50)	1 (50)	1 (50)	1 (50)	1 (50)	1 (50)	1 (50)	1 (50)	1 (50)
Acinetobacter sp (1)	NA	NA	1 (100)	1 (100)	1 (100)	1 (100)	1 (100)	0 (0)	1 (100)	0 (0)
Antibiotic resistance pattern, *n* (%)										

Gram-positive bacteria	P	AMP	AMC	FOX	CN	SXT	E	CD		
*S. aureus* (18)	15 (83.3)	15 (83.3)	12 (66.7)	12 (66.7)	4 (22.2)	14 (77.8)	8 (44.4)	1 (5.6)		
CoNS (13)	11 (84.6)	11 (84.6)	10 (77)	10 (77)	4 (30.8)	7 (53.8)	4 (30.8)	1 (7.7)		

AMP Ampicillin, AMC Amoxicillin-clavulanic acid, CD Clindamycin, CTX Cefotaxime, CAZ Ceftazidime, CPM Cefepime, CRO Ceftriaxone, *E* Erythromycin, FOX Cefoxitine, CN Gentamicin, MEM Meropenem, PTZ Piperacillin-Tazobactam, P Penicillin, SXT Trimethoprim-Sulfamethoxazole.; NA = NotApplicable; CoNS = Coagulase-negative *Staphylococcus* spp.

**Table 6:** Multidrug resistance patterns among isolated bacteria from blood cultures in neonates with suspected sepsis admitted in NICU at Dessie Comprehensive Specialized Hospital, Dessie, Northeastern Ethiopia, August 2017 to March 2018.

Isolated bacteria	Degree of resistance							
	R0	R1	R2	R3	R4	R5	>=R6	Total MDR (≥3 antibiotic classes)
Gram-negative (36)								
*E. coli* (24)	5	1	3	1	1	2	11	15
*K. pneumoniae* (7)	0	0	1	1	0	0	5	6
*K. oxytoca* (2)	0	0	1	0	0	0	1	1
Citrobacter sp (2)	1	0	0	0	0	0	1	1
Acinetobacter sp (1)	0	0	0	0	0	0	1	1
Total	6	1	5	2	1	2	19	24
Gram-positive (31)								

*CoNS* (13)	2	1	0	2	4	0	4	10
*S. aureus* (18)	3	1	2	0	8	0	4	12
Total	5	2	2	2	12	0	8	22

R0: no resistance, R1: Resistance to one antibiotic class, R2 : Resistance to two antibiotic classes, R3 : Resistance to three antibiotic classes, R4 : Resistance to four antibiotic classes, R5 : Resistance to five antibiotic classes.

**Table 7:** Distribution of drug susceptibility pattern of positive blood culture among improved and died neonates admitted in NICU at Dessie Comprehensive Specialized Hospital, Dessie, Northeastern Ethiopia, August 2017 to March 2018.

Outcome	Pattern	Drug susceptibility pattern, (n)												
		*P*	AMP	AMC	PTZ	FOX	CXT	CAZ	CPM	MEM	CN	SXT	*E*	CD
Improved *n* = 61	S	5	11	17	12	9	14	15	17	29	41	20	19	29
	I	31	1	1	29	31	31	30	30	30	0	0	31	31
	R	25	49	43	20	21	16	16	14	2	20	41	11	1
Death *n* = 6	S	0	0	0	2	0	0	0	0	2	2	0	0	0
	I	5	0	0	1	5	1	1	1	1	0	0	5	5
	R	1	6	6	3	1	5	5	5	3	4	6	1	1
Total *n* = 67	S	5	11	17	14	9	15	15	17	31	43	20	19	29
	I	36	1	1	30	36	31	31	31	31	0	0	36	36
	R	26	55	49	23	22	21	21	19	5	24	47	12	2

*R*- Resistance, *I*–Intermediate, *S*—Sensitive, AMP—Ampicillin, AMC- Amoxicillin-clavulanic acid, CD- Clindamycin, CTX- Cefotaxime, CAZ- Ceftazidime, CPM- Cefepime, *E* -Erythromycin, FOX- Cefoxitine, CN—Gentamicin, MEM - Meropenem, PTZ- Piperacillin-Tazobactam, P Penicillin, SXT Trimethoprim-Sulfamethoxazole.

## Data Availability

The authors confirm that all data underlying the findings are fully available without restriction. All relevant data are within the manuscript.

## Abbreviations

AMR: Anti-microbial resistant
ATCC: American type culture collection
CoNS: Coagulase negative *Staphylococcus*
DCSH: Dessie comprehensive specialized hospital
EOS: Early onset neonatal sepsis
ESBL: Extended spectrum beta lactamase
LOS: Late onset neonatal sepsis
MDR: Multidrug Resistant
MRSA: Methicillin resistant *Staphylococcus aureus*
NICU: Neonatal intensive care unit
SPSS: Statistical package for social science
WHO: World health organization
PROM: Prolonged rupture of membrane
MHA: Mueller hinton agar.
